# CD70–CD27 ligation between neural stem cells and CD4^+^ T cells induces Fas–FasL-mediated T-cell death

**DOI:** 10.1186/scrt206

**Published:** 2013-05-21

**Authors:** Eun Mi Lee, Sunghoon Hurh, Bumrae Cho, Kook-Hwan Oh, Seung U Kim, Charles D Surh, Jonathan Sprent, Jaeseok Yang, Jae Young Kim, Curie Ahn

**Affiliations:** 1 Transplantation Research Institute, Seoul National University Medical Research Center, College of Medicine Seoul National University, 103 Daehak-ro, Jongno-gu, Seoul 110-799, Korea; 2 Department of Medicine, Program of Immunology, The Graduate School, College of Medicine Seoul National University, 103 Daehak-ro, Jongno-gu, Seoul 110-799, Korea; 3 Department of Internal Medicine, Seoul National University Hospital, 101 Daehak-ro, Jongno-gu, Seoul 110-744, Korea; 4 Medical Research Institute, Chungang University School of Medicine, 102 Heukseok-ro, Dongjak-gu, Seoul 156-755, Korea; 5 Department of Immunology & Microbial Science, The Scripps Research Institute, La Jolla, CA, USA; 6 Immunology and Inflammation Research Programme, Garvan Institute of Medical Research, Darlinghurst, NSW, Australia; 7 Transplantation Center, Seoul National University Hospital, 101 Daehak-ro, Jongno-gu, Seoul 110-744, Korea; 8 Department of Biological Science, Gachon University of Medicine and Science, 191 Hambangmoe-ro, Yeonsu-gu, Incheon 406-799, Korea

**Keywords:** Neural stem cells, Co-stimulatory molecules, Immune escape mechanism

## Abstract

**Introduction:**

Neural stem cells (NSCs) are among the most promising candidates for cell replacement therapy in neuronal injury and neurodegenerative diseases. One of the remaining obstacles for NSC therapy is to overcome the alloimmune response on NSCs by the host.

**Methods:**

To investigate the mechanisms of immune modulatory function derived from the interaction of human NSCs with allogeneic T cells, we examined the immune regulatory effects of human NSCs on allogeneic T cells *in vitro*.

**Results:**

Significantly, NSCs induced apoptosis of allogeneic T cells, in particular CD4^+^ T cells. Interaction of CD70 on NSCs and CD27 on CD4^+^ T cells mediated apoptosis of T cells. Thus, blocking CD70–CD27 interaction prevented NSC-mediated death of CD4^+^ T cells.

**Conclusions:**

We present a rational explanation of NSC-induced immune escape in two consecutive stages. First, CD70 constitutively expressed on NSCs engaged CD27 on CD4^+^ T cells, which induced Fas ligand expression on CD4^+^ T cells. Second, CD4^+^ T-cell apoptosis was followed by Fas–Fas ligand interaction in the CD4^+^ T cells.

## Introduction

Neural stem cells (NSCs) are among the most promising candidates for cell replacement therapy in neuronal injury and neurodegenerative diseases. Derivation methods to produce neuronal cells with specific functions from NSCs, such as dopaminergic neurons, have also been established [1]. Therefore, it is highly likely that NSCs will become the first choice for stem cell treatment.

Most cellular and organ transplantation between two unrelated individuals results in graft rejection. However, it has been often found that stem cell transplantation could escape the graft rejection [2-4], presumably by inducing apoptosis of T cells via Fas–Fas ligand (FasL) interaction [5].

Transplantation of rodent embryonic stem cells (ESCs) into allogeneic recipients indicated that they may have immune-privileged properties. The injection of undifferentiated rat ESC-like cells into fully MHC-mismatched rats led to the induction of donor-specific immunological tolerance [6]. Moreover, undifferentiated allogeneic murine ESCs led to engraftment of these cells in the thymus, spleen and liver, with establishment of mixed hematopoietic chimerism [7]. This also proved that ESCs represent a good research tool for possible therapeutic applications in solid organ transplantation because of their capability, when given alone, to induce tolerance to semi-allogeneic solid organ allograft [8].

NSC transplantation could be applied to the brain because this organ is subject to less surveillance by the immune system than other parts of the body, and like brain cells NSCs may display characteristics of cells with immune privilege. Consistent with this idea, T cells that infiltrate around transplanted NSCs in a stroke model exhibited little alloimmune response [9], suggesting NSCs have a strong potential to protect cells of the central nervous system against harmful T-cell infiltration [10]. In a related study, the systemic injection of neuronal stem/precursor cells provided a remarkable amelioration of the pathological features of experimentally induced autoimmune encephalomyelitis and other preclinical models of neurological disorders [11,12].

In this paper, we have investigated the mechanisms responsible for NSC-induced immune tolerance. Our studies indicate a two-stage model whereby human NSCs induce apoptosis of allogeneic CD4^+^ T cells. First, CD70 expressed constitutively on NSCs engage CD27 on CD4^+^ T cells and induce upregulation of FasL expression on the CD4^+^ T cells. Second, T cells thereafter undergo apoptosis from fratricide; that is, through Fas–FasL interaction in the CD4^+^ T cells themselves.

## Materials and methods

### Cell culture

The immortalized human embryonic neural stem cell line, HB1.F3 was generated in a previous study by transfection of primary cells from fetal human telencephalon tissue of 14 weeks gestation with amphotropic, replication-incompetent retroviral vector containing *v-myc*[13]. Primary NSCs were provided by Professor Kim (Korea University, Seoul, Korea) [14,15]. HB1.F3 cells and primary NSCs were cultured in DMEM with 10% heat-inactivated fetal bovine serum (Gibco, Carlsbad, CA, USA), and 1% antibiotic–antimycotic solution (Gibco, Carlsbad, CA, USA). Human umbilical cord blood mesenchymal stem cells purchased from MEDIPOST Co., Ltd (Seoul, Korea) were used. Human umbilical cord blood-derived mesenchymal stem cells were isolated and expanded as described previously [16,17]. Cultures were maintained in a humidified incubator at 37°C containing 5% CO_2_.

### CD4^+^ and CD8^+^ T-cell preparation

Whole blood was collected from healthy volunteer donors (*n* = 55, age: 24.85 ± 3.52, sex: male/female = 36/19). The institutional review board of the Seoul National University Hospital approved this study, and all volunteers provided written informed consent. Peripheral blood mononuclear cells were isolated by Ficoll-Paque PLUS (Amersham Biosciences, Piscataway, NJ) density gradient centrifugation of peripheral venous blood [18]. The peripheral blood mononuclear cells were separated into CD4^+^ and CD8^+^ T-cell populations by magnetic-activated cell separation (Miltenyi Biotec, Bergisch Gladbach, Germany).

### Co-culturing T cells with neural stem cells

To determine NSC-dependent apoptosis, CD4^+^ or CD8^+^ T cells were cultured with HB1.F3 at a density of 5 × 10^4^:5 × 10^3^, 5 × 10^4^:1 × 10^4^, 5 × 10^4^:2.5 × 10^4^, 5 × 10^4^:5 × 10^4^, and 5 × 10^4^:1 × 10^5^ cells/well on 96-well plates in well media. Adherent NSCs and nonadherent T cells were separately harvested at the indicated times and washed in 1× PBS.

### Analysis of T-cell apoptosis

Apoptotic cell death was assessed using Annexin V staining coupled with propidium iodide (BD PharMingen, San Diego, CA, USA). T cells were cultured with HB1.F3 at a various densities or times. T cells were washed twice with 1× PBS. Then 2 × 10^5^ to 5 × 10^5^ T cells were added to a 12 mm test tube and washed twice in 1 ml of 1× binding buffer. Cells were mixed gently with 5 μl Annexin V–fluorescein isothiocyanate and incubated at 4°C in the dark for 5 minutes. After washing with binding buffer, 10 μl of the 20 μg/ml propidium iodide solution (end concentration 1 μg/ml) and 190 μl binding buffer were added to the cells. Cells were immediately analyzed by fluorescence-activated cell sorting (FACS).

### Flow cytometry

Adherent NSCs were trypsinized and resuspended in staining buffer (0.1% BSA in PBS) to reach a final concentration of 5 × 10^5^ to 1 × 10^6^ cells/ml. The cells were incubated for 20 minutes on ice with the following antibodies: anti-CD27 (O323), anti-CD70 (Ki-24), anti-Fas (APO-1), anti-FasL (NOK-1), anti-PD-1 (J105), anti-PD-L1 (MIH1), anti-TR-1 (DJR1), anti-TR-2 (DJR2-4), and anti-TRAIL (RIK-2). After incubation, the cells were washed twice with staining buffer and resuspended. The stained cells were analyzed by FACS.

### Semiquantitative RT-PCR

Total RNA was prepared from CD4^+^ T cells using the RNA isolation kit (QIAGEN Inc, Valencia, CA, USA). Two micrograms of RNA were reverse-transcribed. The reaction mixture consisted of 20 mM Tris–HCl (pH 8.4), 50 mM KCl, 5 mM MgCl_2_, 10 mM dithiothreitol, 40 U of RNaseOUT™, 1 mM dNTP, 2.5 μM of oligo(dT)_20_, 200 U of SuperScript™ III reverse transcriptase (Invitrogen, Carlsbad, CA, USA) and the remaining volume was diethylpyrocarbonate-treated water for a total volume of 20 μl. The mixture was incubated at 50°C for 1 hour, and at 85°C for 5 minutes. For semiquantitative PCR, the reaction mixture consisted of 10 mM Tris–HCl, 50 mM KCl, 0.1% Triton X-100, 2 mM MgCl_2_, 0.4 mM dNTP, 0.2 pmol sense and antisense primer, 2.5 U Taq DNA polymerase and 3 μg cDNA. Primer sequences are provided in Additional file 1.

### HLA-A, HLA-B and HLA-DR allele on HB1.F3 cells

We examined HLA-A, HLA-B, and HLA-DR alleles on HB1.F3 cells using the PCR sequence-specific oligonucleotide method (Dynal RELI™ HLA typing kit, Dynal Biotech, Wirral, U.K.) [19]. The results are provided in Additional file 2.

### Treatment of anti-human Fas ligand mAbs

CD4^+^ T cells were incubated with HB1.F3 for 24 hours with a series of concentrations of neutralizing Fas ligand mAb (NOK-2): 0, 0.01, 0.1, 0.5, and 1 μg/ml.

### Treatment of actinomycin D and cycloheximide

We treated a transcription inhibitor, actinomycin D (1 μg/ml; Sigma-Aldrich, St. Louis, MO, USA), on CD4^+^ T cells for 1 hour before co-culture with HB1.F3. The cells were co-cultured for 0 to 18 hours, and death ligand on CD4^+^ T cells was assessed at the transcriptional level by RT-PCR. We treated a translation inhibitor, CHX (10 μg/ml, Sigma-Aldrich, St. Louis, MO, USA) on T cells for 12 hours before co-culture with HB1.F3. The cells co-cultured for 12 hours, and intracellular Fas ligand expression on CD4^+^ or CD8^+^ T cells and HB1.F3 was assessed by FACS.

### Treatment of anti-human CD27 or CD70 mAbs

CD4^+^ T cells were incubated with HB1.F3 for 12 hours with a series of concentrations of anti-CD27 (LG.7F9) or anti-CD70 mAb (BU69): 0, 0.01, 0.1, 0.5, and 1 μg/ml.

### Combinatory treatment of anti-CD27, anti-PD-L1, or anti-CD40 mAb

CD4^+^ T cells were incubated with HB1.F3 for 24 hours with 0.5 μg/ml of anti-CD27 (LG.7F9), anti-PD-L1 (MIH-1), or anti-CD40 mAb (5C3).

### Statistical analysis

All results are expressed as the mean ± standard deviation. The statistical significance of differences between group means was determined using Student’s *t* test.

## Results

### Human neural stem cells induce CD4^+^ T-cell apoptosis

**Figure 1:**
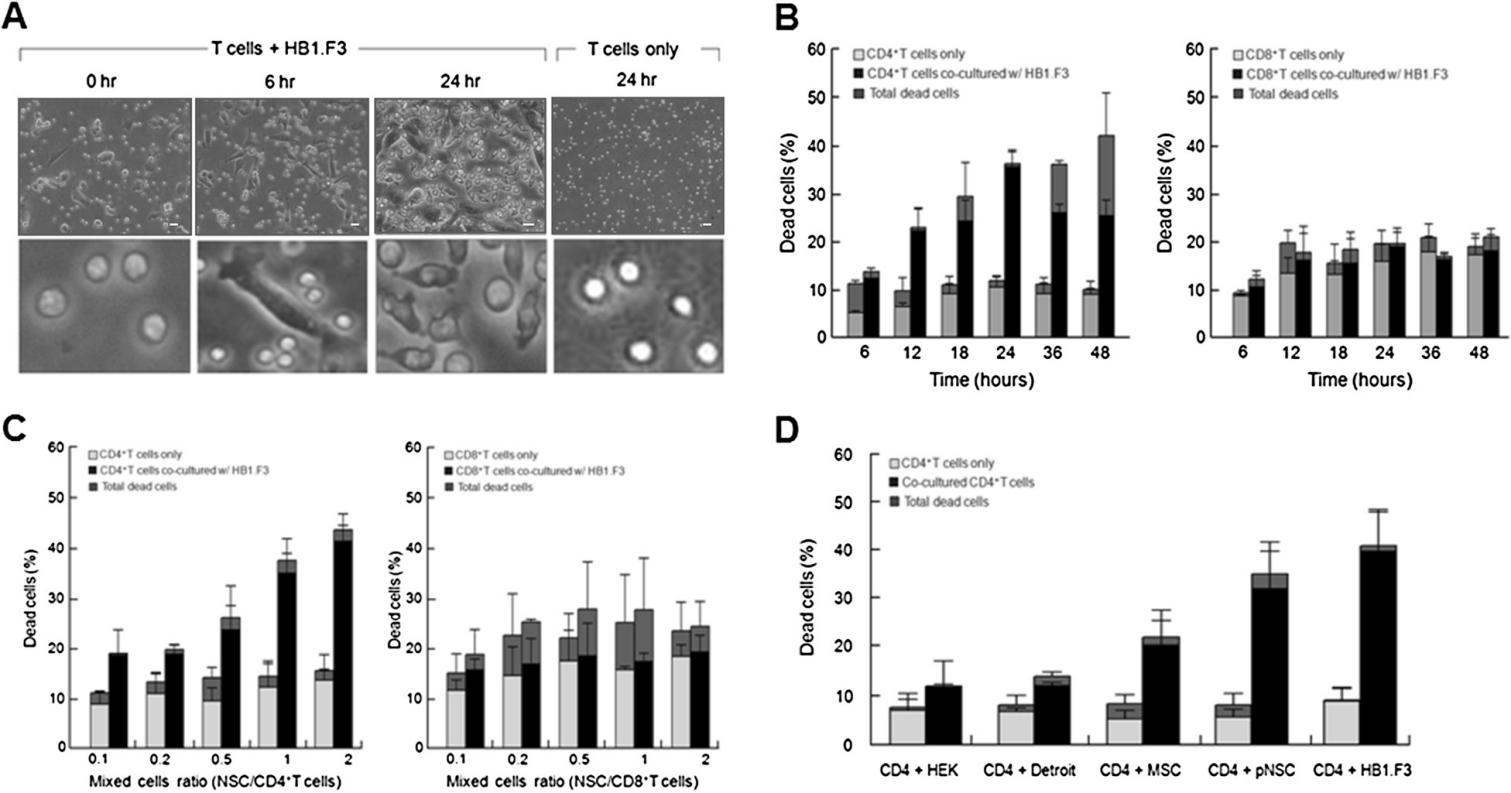
**Human neural stem cells (HB1.F3) induce T-cell apoptosis. (A)** The morphology of CD4^+^ T cells after the co-culture with HB1.F3 was characteristic of apoptotic cells: blebbing and shrinkage of cytoplasm (scale bar: 20 μm). (**B**) CD4^+^ T cells showed maximal apoptosis at 24 hrs (■, AV^+^/PI^-^ and AV^+^/PI^+^ cells), however the total dead cells of T cells increased by time dependent manner (, total of AV^+^/PI^-^, AV^+^/PI^+^, and AV^-^/PI^+^ cells). **(C)** The degree of CD4^+^ T-cell apoptosis occurred in an HB1.F3 density-dependent manner. **(D)** Contrary to CD4^+^ T-cell apoptosis by pNSCs or HB1.F3, the apoptosis levels of CD4^+^ T cells by HEK-293, Detroit 551, and human umbilical cord blood-derived mesenchymal stem cells did not significantly differ from each other. MSC, mesenchymal stem cell; NSC, neural stem cell.

To assess the extent of allogeneic response against NSCs, the *in vitro* response of human T cells was measured towards the fetal NSC line HB1.F3 [20]. Surprisingly, the majority of human T cells displayed morphology of apoptotic cells within 24 hours upon incubation with HB1.F3 (Figure 1A). Apoptosis of T cells commenced within 6 to 12 hours and reached the maximum at 24 hours after co-culturing with HB1.F3 (Figure 1B). The induction of cell death was prominent for CD4^+^ T cells, affecting ~30 to 40% above the background, but was negligible for CD8^+^ T cells (Figure 1B). The extent of CD4^+^ T-cell death increased with a higher ratio of HB1.F3 to T cells, while the extent of CD8^+^ T-cell apoptosis did not rise with elevated HB1.F3 ratio (Figure 1C). In addition to HB1.F3, primary NSCs induced CD4^+^ T-cell apoptosis. NSCs appear unique in their ability to induce apoptosis of CD4^+^ T cells, because other types of cells, including fibroblasts, epithelial cells, and even stem cells of another lineage (mesenchymal stem cells), did not induce apoptosis of CD4^+^ T cells (Figure 1D).

### Fas–Fas ligand interaction is involved in neural stem cell-induced T-cell apoptosis

**Figure 2:**
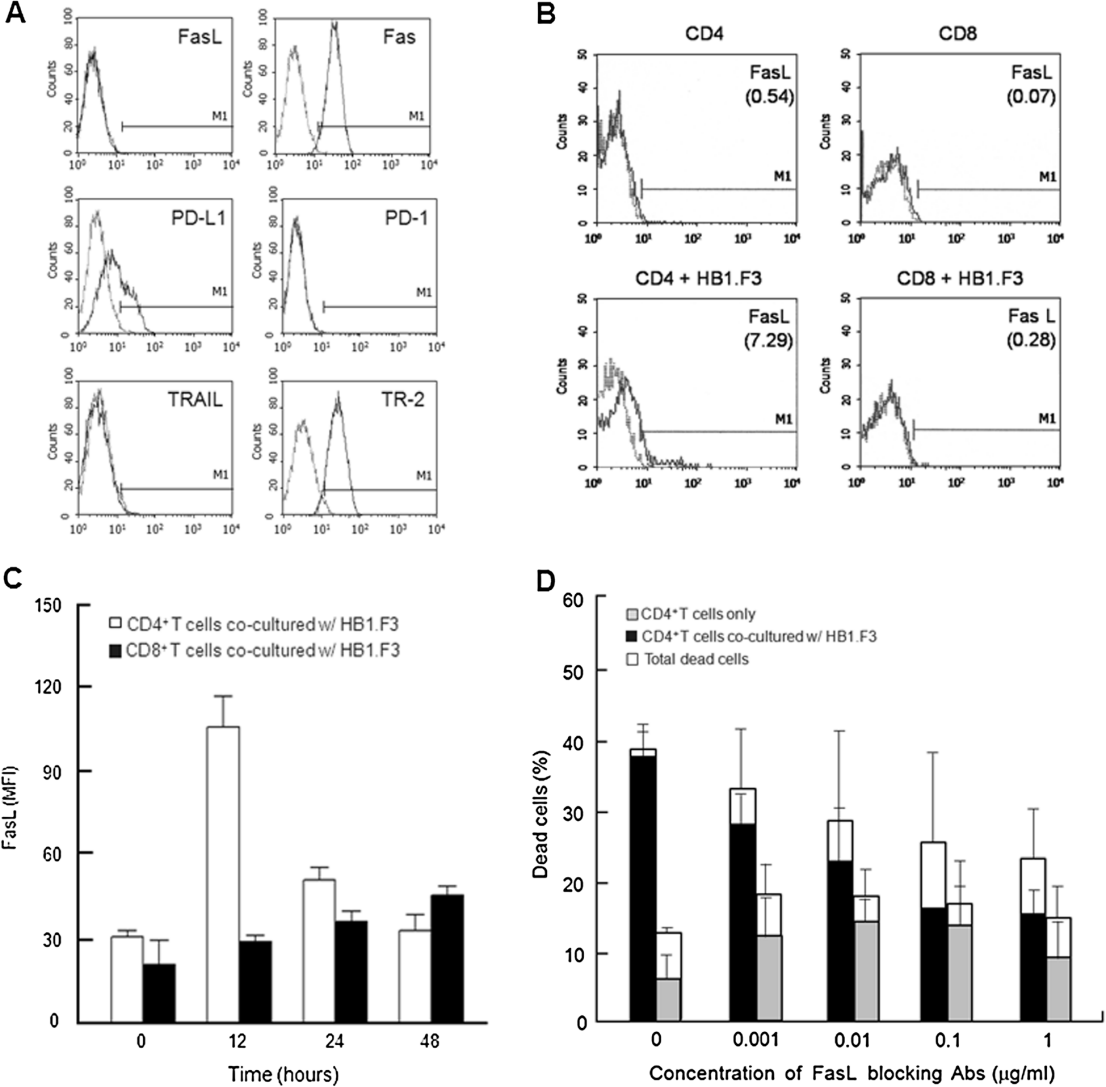
**Effect of Fas ligand expression on HB1.F3-induced T-cell apoptosis. (A)** Fas, PD-L1, and TR-2 (TRAIL receptor-2) were expressed on HB1.F3 cells. **(B)** CD4^+^ T cells, CD8^+^ T cells, and HB1.F3 cells constitutively did not express Fas ligand (FasL). FasL expression on CD4^+^ T cells co-cultured with HB1.F3 was induced at 12 hours, but FasL expression on CD8^+^ T cells was much less dramatic. The FasL level of CD4^+^ T cells was higher than CD8^+^ T cells in a small population. **(C)** FasL (median fluorescence intensity) on CD8^+^ T cells did not significantly change (dark shading) during the co-culture period, whereas marked FasL upregulation on CD4^+^ T cells was detected (no shading). (**D**) The inhibition of CD4^+^ T cells apoptosis showed anti-FasL mAbs dose-dependent manner.

To determine the mechanism of T-cell apoptosis mediated by NSCs, we analyzed for expression of death-inducing molecules Fas, FasL, PD-1, PD-L1, TRAIL receptor-1, TRAIL receptor-2, and TRAIL on HB1.F3, as these molecules were previously reported to be present on stem cells [21-24]. HB1.F3 cells expressed high levels of Fas and TRAIL receptor-2 on cell surface, but not FasL, TRAIL, and PD-1 (Figure 2A). Since human PBL do not express FasL [25], T cells presumably upregulated FasL in order to be susceptible to Fas-mediated cell death by NSCs. To confirm this notion, FasL expression on T cells was analyzed after co-culture with HB1.F3 cells. FasL expression on the cell surface was slightly upregulated on the majority of CD4^+^ T cells and a small fraction (~7.3%) of cells expressed high levels of FasL (109.96 ± 11.52) (Figure 2B,C). The peak of FasL upregulation was at 12 hours post incubation with HB1.F3. In contrast to CD4^+^ T cells, FasL upregulation was minimal on CD8^+^ T cells (Figure 2C). After blocking of Fas-FasL interaction with an anti-FasL mAb (NOK-2) inhibited NSCs-induced CD4^+^ T cell apoptosis in a dose-dependent manner (Figure 2D).

### Neural stem cells induce Fas ligand upregulation on T cells

**Figure 3:**
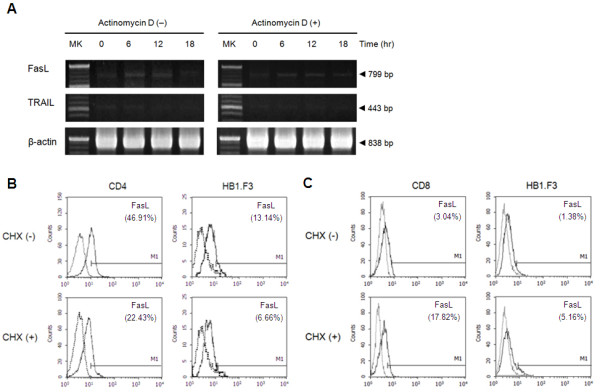
**The effect of Actinomycin D and cycloheximide on death ligand expression levels on CD4**^**+**^** T cells and HB1.F3.** (**A**) Actinomycin D (Act D) abolished induction of Fas ligand (FasL) transcription on CD4^+^ T cells at 12 hours, but TRAIL has no differences in the co-culture period. (**B**), (**C**) Intracellular FasL expression levels on co-cultured CD4^+^ T cells and CD8^+^ T cells, and HB1.F3 without cycloheximide (CHX−) and with cycloheximide (CHX+). Intracellular FasL expression on CD4^+^ T cells and co-cultured HB1.F3 were reduced about 50% (46.91 vs. 22.43, 13.14 vs. 6.66 (**B**)). On the other hand, intracellular FasL of CD8^+^ T cells and co-cultured HB1.F3 were increased slightly (3.04 vs. 17.82, 1.38 vs. 5.16 (**C**)).

Induction of FasL was evident also at the mRNA level in CD4^+^ T cells within 12 hours after interaction with HB1.F3 cells (Figure 3A). FasL mRNA upregulation was blocked by actinomycin D, indicating new synthesis of FasL (Figure 3A). Figure 3B and C shows the intracellular FasL expression levels on co-cultured CD4^+^ T cells and CD8^+^ T cells, and HB1.F3 without cycloheximide and with cycloheximide. Intracellular FasL upregulation was higher on CD4^+^ T cells than CD8^+^ T cells. Upregulation of FasL on CD4^+^ T cells was blocked by cycloheximide, but not CD8^+^ T cells. Intracellular FasL expression on CD4^+^ T cells and co-cultured HB1.F3 were reduced about 50% (46.91 vs. 22.43, 13.14 vs. 6.66; Figure 3B). On the other hand, intracellular FasL expression on CD8^+^ T cells and co-cultured HB1.F3 was increased slightly (3.04 vs. 17.82, 1.38 vs. 5.16; Figure 3C). Cycloheximide is assumed to affect the Fas-induced apoptosis on CD8^+^ T cells and HB1.F3.

### CD70–CD27 interaction is involved in neural stem cell-induced CD4^+^ T-cell death

**Figure 4:**
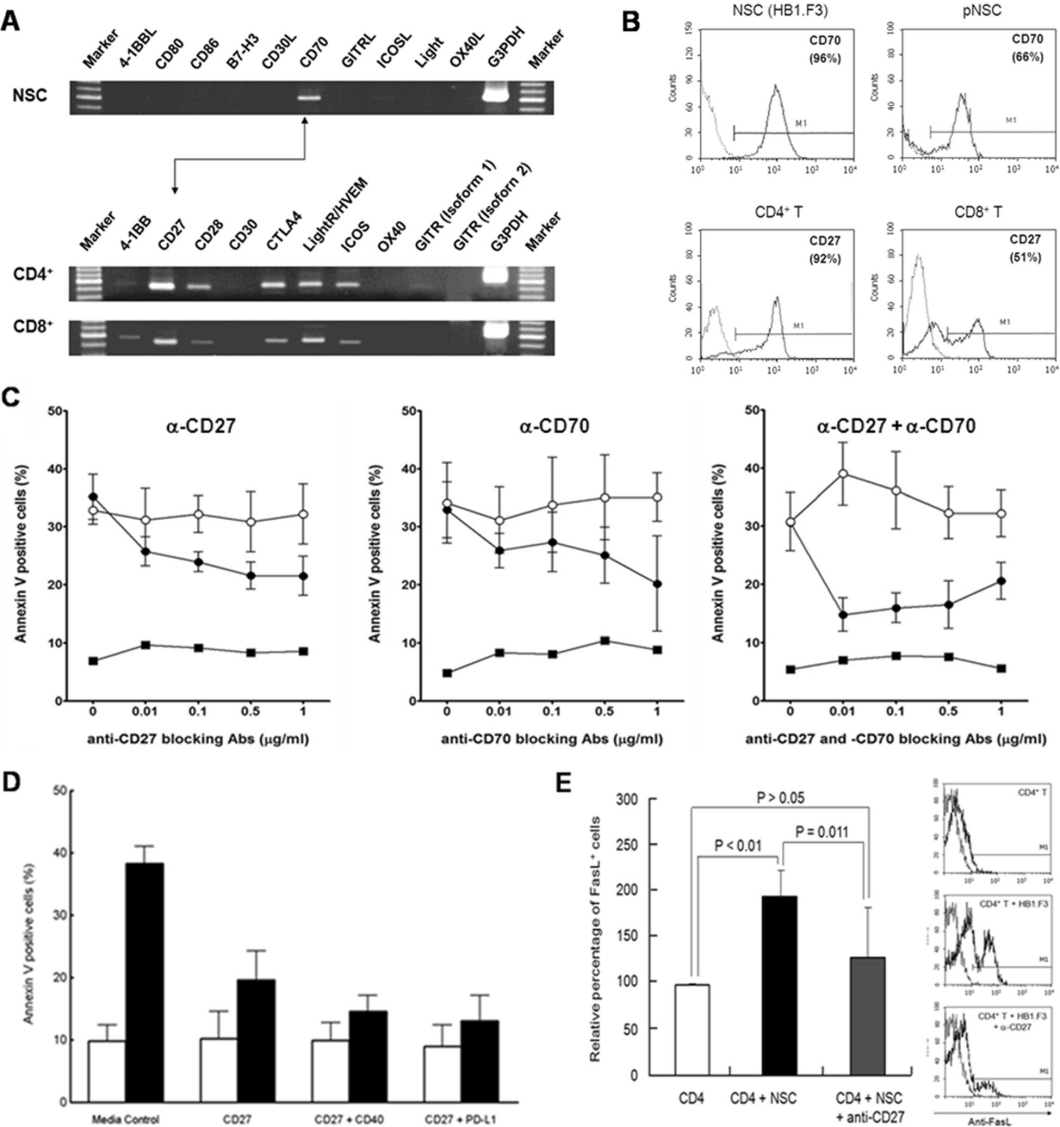
**CD70-CD27 interaction was involved in HB1.F3-induced T cell apoptosis.** (**A**) While constitutive expression of CD70 mRNA was detected on HB1.F3, other co-signaling molecule mRNA was not detected. T cells constitutively expressed CD27 as well as several the other co-signaling molecules such as CD28, CTLA4, HVEM, and ICOS. **(B)** CD70 expression on HB1.F3 and CD27 on T cells was also confirmed by fluorescence-activated cell sorting (FACS). CD27 expression level on CD4^+^ T cells were above 90%, but CD8^+^ T cells were about 50%. **(C)** Addition of anti-CD27 and CD70 mAbs resulted in a dose-dependent inhibition of the CD4^+^ T-cell apoptosis but did not inhibit the apoptosis completely (■, CD4^+^ T cells only; ○, CD4^+^ T cells co-cultured w/ HB1.F3 + isotype control mAbs); ●, CD4^+^ T cells co-cultured w/ HB1.F3 + anti- CD27 or anti-CD70 mAbs). **(D)** To assess the impact of anti-CD40 and PD-L1 mAbs on the CD4^+^ T-cell apoptosis, we measured the CD4^+^ T-cell apoptosis ratio after anti-CD27, anti-CD40 or anti-PD-L1 mAbs treatment. This showed an inhibitory effect of the CD4^+^ T-cell apoptosis compared with the media control group (□, CD4^+^ T cells only; ■, CD4^+^ T cells w/ HB1.F3). (**E**) FasL expression level on CD4^+^ T cells reduced about 50% by addition of anti-CD27 mAb.

To determine the molecular interactions responsible for induction of FasL on CD4^+^ T cells, we assessed expression of several co-stimulatory molecules on HB1.F3 and T cells by RT-PCR. HB1.F3 constitutively expressed CD70 mRNA, but not other co-stimulatory molecules, including 4-1BBL, CD80, CD86, ICOSL, and OX40L (Figure 4A). The expression pattern of these co-stimulatory molecules on NSCs was confirmed by FACS analysis, which showed that the majority of both HB1.F3 and primary NSCs expressed CD70, but not other molecules (Figure 4B and data not shown). As reported previously [26], freshly purified human CD4^+^ and CD8^+^ T cells were found to express mRNA for CD27, the receptor for CD70, and other co-stimulatory molecules, including CD28, HVEM and ICOS (Figure 4A). In addition, as reported previously [27], CD27 expression was found on nearly on all human CD4^+^ T cells, but only ≈ 50% on CD8^+^ T cells (Figure 4B). The presence of CD70 on NSCs and CD27 on T cells led us to consider the possibility that CD70–CD27 interaction could play an important role in NSC-induced apoptosis of the T cells. To this end, the effect of anti-CD27 or anti-CD70 blocking antibodies was evaluated. The presence of either blocking antibody inhibited NSC-mediated death of CD4^+^ T cells in a dose-dependent manner, but only at ~50% of the level, with anti-CD70 blocking antibodies mediating a slightly stronger inhibition (Figure 4C). A synergistic effect of the two blocking antibodies was observed as the presence of both blocking antibodies did lead to an enhanced, but incomplete, inhibition of apoptosis (Figure 4C). Otherwise, we found the expression of PD-L1 on HB1.F3 (as in Figure 2A). In a related study, PD-L1-induced effector T-cell apoptosis was known as a mechanism of immune privilege of corneal allografts [28]. The CD40 system also regulates apoptosis, positively or negatively, depending on the target cells. We therefore confirmed whether PD-L1 and CD40 were involved in T-cell apoptosis by HB1.F3. Figure 4D shows the inhibitory effects by the addition of anti-CD40 or anti-PD-L1 mAb with anti-CD27 mAb. Also, after treatment with anti-CD27 mAbs, the number of FasL-positive CD4^+^ T cells was markedly reduced to the baseline level (Figure 4E).

## Discussion

Immune escape induced by stem cells is partly due to reduced expression of several cell surface molecules of immune activation on stem cell surfaces. For example, reduced alloreactivity caused by lack of MHC II and reduced MHC I expression on ESCs has been reported [29]. Others have reported that stem cells induced T-cell apoptosis by Fas–FasL interaction, which had been observed to be one of the immune escape mechanisms on T cells [5,30].

CD4^+^ T-cell apoptosis seemed to continuously occur even 24 hours after the co-culture, because the number of propidium iodide-positive cells continuously increased even after the number of Annexin V-positive cells reached its peak at 24 hours. However, it has not been clearly demonstrated whether this reaction was caused by direct interaction between the FasL expressed on the surface of NSCs and Fas on CD4^+^ T cells. Similar to other researchers' observations of human ESCs in various stages of differentiation [2], we could not detect either FasL mRNA or surface protein by FACS in NSCs. FasL expression on HB1.F3 was examined using FACS analysis and immunocytochemistry. Both results showed an absence of FasL on HB1.F3, which was similar to the previous results of a study performed with ESCs [29]. Direct Fas–FasL interactions between NSC and CD4^+^ T cells are therefore less likely to occur (see Additional file 3). NSCs must therefore have provoked Fas–FasL-mediated CD4^+^ T-cell apoptosis through an indirect pathway.

In addition, transplant rejection occurs through recognition of foreign antigens presented by MHC molecules expressed on antigen-presenting cells, a process that can be mimicked by triggering T cells with CD3-specific mAb [31]. We used anti-CD3 (OKT3) and anti-CD28 (CD28.2) mAbs, and recombinant mouse CD27L (Minneapolis, MN, USA) to stimulate T cells in a manner that partially mimics stimulation by antigen-presenting cells. We then checked FasL expression and apoptosis of CD4^+^ T cells. Our previous data showed an increase of FasL-positive cells and apoptotic CD4^+^ T cells by antigen presentation by T-cell receptor and co-stimulatory signals. However, the median fluorescence intensity of FasL and the apoptosis level varied by blood donor, incubation time, and the combinations of antibodies and recombinant protein (see Additional file 4).

In this paper, we demonstrated that CD70–CD27 interaction was involved in NSCs-induced CD4^+^ T cell apoptosis. Originally, CD27 is a cell surface glycoprotein belonging to the TNF receptor superfamily, which can provide stimulatory signals for both cell growth and apoptosis. CD70–CD27 interactions between most immune cells produce T cell expansion and the development of effector cytotoxic or memory T cells [32]. On the other hand, it has been reported that CD27 can bind apoptosis-inducing factor (Siva 1), an intracellular mediator of apoptosis [33,34], but the role of this interaction for the function of CD27 is yet to be resolved, because ligation of CD27 generally does not limit but rather contributes to the expansion of activated lymphocytes [35].

CD70 expressed on malignant cells showed novel function in immune escape [36]. Human brain tumor cells, such as malignant glioma like or glioblastoma multiforme, express CD70 [37], and this CD70 – but not TNFα or FasL – initiated T-cell death through the receptor-dependent pathway [38]. However, the exact mechanism of this process is not yet fully understood.

Levels of inhibition by both antibodies were otherwise incomplete, implicating the existence of other causes for apoptosis in addition to CD70–CD27 interaction. In CD70-mediated T-cell apoptosis, the role of Siva, a pro-apoptotic molecule, was identified, as well as soluble mediators such as transforming growth factor beta [32,39]. We evaluated the expression of Siva protein using western blotting. Siva was increased at 48 hours in CD4^+^ T cells and the NSC co-culture system. We therefore think that, at least in our system, apoptosis was mainly induced by the FasL upregulation by CD27–CD70 ligation between NSCs and CD4^+^ T cells in the early phase (see Additional file 5).

We then examined cytokine profiles from the supernatant at various co-culture times (1, 3, 6, 12, 18, and 24 hours). Several cytokines (such as IL-1β, TNFα, IL-4, IL-10, IFNγ, IL-5, and IL-13) were shown to be negative in the co-culture periods. Moreover, IL-6 increased in a time-dependent manner. However, the IL-6 level is similar in the NSC-only culture group. In addition, the supernatant of the CD4^+^ T-cell-only culture group was shown to be negative for IL-6. Therefore, IL-6 might require NSC self-renewal and progenitor cell division and differentiation [40-42]. As in ESCs, lack of co-stimulation [9,43] or participation of soluble mediators could play a role in T-cell immune escape. However, activation-induced cell death was less likely to play a role [44] since CD4^+^ T-cell apoptosis in our experiments reached its peak in 24 hours.

Finally, we have demonstrated that FasL expression on CD4^+^ T cells was significantly increased as a consequence of CD70–CD27 ligation. Antibody blocking experiments also confirmed that FasL expression on CD4^+^ T cells was CD70–CD27 dependent. NSCs therefore probably induce CD4^+^ T-cell apoptosis in two stages: CD70–CD27 ligation between NSCs and CD4^+^ T cells, which induces FasL expression on some CD4^+^ T cells, followed by Fas–FasL-mediated CD4^+^ T-cell apoptosis.

## Conclusion

We demonstrated CD70–CD27 ligation to be an initiating step for NSC-induced CD4^+^ T-cell apoptosis. NSCs probably induce CD4^+^ T-cell apoptosis in two stages: CD70–CD27 ligation between NSCs and CD4^+^ T cells, which induces FasL expression on some CD4^+^ T cells, followed by Fas–FasL-mediated CD4^+^ T-cell apoptosis. Therefore, if it is possible to maintain persistent expression of CD70 using a gene delivery technique, this will be an exciting option to maintain immune escape in NSC transplantation.

## Abbreviations

BSA: Bovine serum albumin; DMEM: Dulbecco’s modified eagle’s medium; ESC: Embryonic stem cell; FACS: Fluorescence-activated cell sorting; FasL: Fas ligand; IFN: Interferon; IL: Interleukin; mAb: Monoclonal antibody; MHC: Major histocompatibility complex; NSC: Neural stem cell; PBS: Phosphate-buffered saline; PCR: Polymerase chain reaction; RT: Reverse transcriptase; TNF: Tumor necrosis factor.

## Competing interests

The authors declare that they have no competing interests.

## Authors’ contributions

EML was responsible for conception and design, *in vitro* cell work, collection and assembly of data, manuscript writing, and final approval of the manuscript. SH was responsible for RT-PCR analysis and interpretation of data. BC was responsible for western blot analysis and interpretation of the data. K-HO was responsible for review design and interpretation of the data. SUK was responsible for material support (HB1.F3). CDS was responsible for manuscript writing and review, interpretation of the data, and redesign research. JS was responsible for review of the manuscript, interpretation of the data, and redesign research. JY was responsible for review design, interpretation of the data, and redesign research. JYK was responsible for conception and design, interpretation of the data, and manuscript writing, CA was responsible for conception and design, interpretation of the data, manuscript writing, and final approval of manuscript. EML and CA were responsible for the final manuscript and all authors read and approved the final manuscript.

## Supplementary Material

Additional file 1 a table presenting primer sequences for the RT-PCR. Click here for file

Additional file 2 a table presenting phenotype and gene frequencies of HLA-A, HLA-B, HLA-DR loci on HB1.F3 cells defined by DNA typing method. Click here for file

Additional file 3 **a figure showing FasL expression on CD4**^**+**^** T cells (left) and apoptosis of CD4**^**+**^** T cells (right) increased by anti-CD3 (OKT-3, 1 μg/ml; eBioscience, San Diego, CA, USA) and anti-CD28 (CD28.2, 1 μg/ml; BD Pharmingen, San Diego, CA, USA) agonistic antibodies, and recombinant CD27L (1 μg/ml; R&D Systems) treatment (no shading, CD4**^**+**^** T + anti-CD3; solid shading, CD4**^**+**^** T + msCD27L; vertical shading, CD4**^**+**^** T + anti-CD28; diagonal shading, CD4**^**+**^** T + anti-CD3 + msCD27L; diagonal crosshatch, CD4**^**+**^** T + anti-CD3 + msCD27L + anti-CD28).** The experiment shown is representative of three independent experiments with three different blood donors. Click here for file

Additional file 4 **a figure showing expression of FasL on co-cultured NSCs with allogeneic T cells was examined using FACS analysis (NOK-1) and immunocytochemistry (G247-4).** (**A**) NSCs constitutively did not express FasL. We were able to check the expressions of FasL after IL-1β treatment on NSCs (positive control). (**B**) FasL expression on co-cultured NSCs with allogeneic T cells was not detected. Click here for file

Additional file 5 **a figure showing expression of Siva on co-cultured T cells with NSCs.** Co-cultured CD4^+^ T-cell lysate was tested with anti-Siva antibodies (clone C-20; Santa Cruz, CA, USA) by western blotting. α-tubulin was used as a loading control. Click here for file
