# Supplementary material for: CD70–CD27 ligation between neural stem cells and CD4+ T cells induces Fas–FasL-mediated T-cell death
Source: Stem Cell Res Ther. 2013 May 21;4(3):56. doi: 10.1186/scrt206 (PMC3706991; doi:10.1186/scrt206)
Supplement: Additional file 1 — a table presenting primer sequences for the RT-PCR. [file scrt206-S1.docx]

Table 1. Primer sequences for the RT-PCR.

| primer | Sequences (5' → 3')  (S = sense primer, A = antisense primer) | | | | product size (bp) |
| --- | --- | --- | --- | --- | --- |
| 4-1BBL | S | atg gaa tac gcc tct gac gct tc | A | cag gtg cag cgc aag tga aac | 501 |
| CD80 | S | gga gaa gaa aat ggt gct gac ta | A | tgg ttg tat tcc agt tga agg tc | 500 |
| CD86 | S | acg gtt acc cag aac cta aga ag | A | tgt atc act ttt gtc gca tga ag | 500 |
| PD-L1 | S | cat ttg ctg aac gca ttt act gtc | A | gtg gtc tta cca ctc agg act tg | 500 |
| B7-H3 | S | cta ctc gaa gcc cag cat gac | A | ttt gct gtc aga gtg ttt cag | 502 |
| CD30L | S | cca cta tta tgg tgt tgg tcg ttc | A | cat tct caa gag gaa agg tgc ttg | 507 |
| CD70 | S | gct gct ttg gtc cca ttg gtc | A | cac tgc act cca aag aag gtc tc | 509 |
| GITRL | S | cac ttg gaa aat atg cct tta agc | A | ggg gat ttg cta gta aaa tga tac | 508 |
| ICOSL | S | cgt ata ttg gca aac cag tga gtc | A | ctc tat gca gca gcc aat gtt cac | 501 |
| LIGHT | S | cag acc gac atc cca ttc acg ag | A | gct gac caa cag ctc cag ctc | 501 |
| OX40L | S | caa gat tcg aga gga aca agc tat | A | gac aca gaa ttc acc agg att ttg | 497 |
| 4-1BB | S | cag tgt aaa ggt gtt ttc agg ac | A | tat aca gga gtt tct ttc tgc cc | 469 |
| CD27 | S | cac tgt aac tct ggt ctt ctc gt | A | aca gga ctt tct cct ttg ttt g | 422 |
| CD28 | S | aca atc tct tct caa ggg agt tc | A | ctc tta ctc ctc acc cag aaa at | 418 |
| CD30 | S | agc ctg act act acc tgg atg a | A | gga cag acc tgg atc tga act a | 476 |
| CTLA4 | S | gcc agc ttt gtg tgt gag tat | A | ttc aca tag acc cct gtt gta ag | 449 |
| ICOS | S | gcc tct ggt att tct ttc tct tc | A | aca tcc tat ggg taa cca gaa ct | 431 |
| OX40 | S | act gtg tcg ggg aca cct acc c | A | tgt cct cac aga ttg cgt ccg | 417 |
| PD-1 | S | aga gct tcg tgc taa act ggt a | A | ata gtc cac aga gaa cac agg c | 488 |
| GITR(1) | S | gag gag tgc tgt tcc gag tgg | A | tcc tca gct gcc aga tgt gca g | 391 |
| GITR(2) | S | gag gag tgc tgt tcc gag tgg | A | ctt cct gca ccc act tct gct gc | 644 |
|  |  |  |  |  | 665 |
| GAPDH | S | aca gcc tca aga tca tca gca at | A | agg aaa tga gct tga caa agt gg | 513 |
| FasL | S | atg cag cag ccc ttc aat tac cc | A | cca gag aga gct cag ata cgt tg | 799 |
| Fas | S | tgg cat caa ctt cat gga aa | A | aaa cat cct tgg agg cag aa | 347 |
| TRAIL | S | caa ctc cgt cag ctc gtt aga aag | A | tta gac caa caa cta ttt cta gca ct | 304 |
|  |  |  |  |  | 452 |
| β-actin | S | atc tgg cac cac acc ttc tac aat gag ctg cg | A | cgt cat act cct gct tgc tga tcc aca tct g | 838 |
