# Supplementary material for: CD70–CD27 ligation between neural stem cells and CD4+ T cells induces Fas–FasL-mediated T-cell death
Source: Stem Cell Res Ther. 2013 May 21;4(3):56. doi: 10.1186/scrt206 (PMC3706991; doi:10.1186/scrt206)
Supplement: Additional file 2 — a table presenting phenotype and gene frequencies of HLA-A, HLA-B, HLA-DR loci on HB1.F3 cells defined by DNA typing method. [file scrt206-S2.docx]

Table 2. Phenotype and gene frequencies of HLA-A, -B, -DR loci on HB1.F3 cells defined by DNA typing method.

| Name | HB1.F3 cells (Canadian) | *Gene Frequency in Korean |
| --- | --- | --- |
| HLA-A | A1 (*01)  A31 (*31) | 1.9 %  5.6 % |
| HLA-B | B7 (*07)  **B63 (*15:17)** | 4.4 %  **0.1 %** (very rare in Korean) |
| HLA-DRB1 | DR10 (*10)  DR13 (*13) | 1.7 %  11.0 % |

*Reference: Roh EY, Kim HS, Kim SM et al., Korean Journal of Laboratory Medicine 2003; 23: 420-430.
