# Supplementary figures and images for: CD70–CD27 ligation between neural stem cells and CD4+ T cells induces Fas–FasL-mediated T-cell death
Source: Stem Cell Res Ther. 2013 May 21;4(3):56. doi: 10.1186/scrt206 (PMC3706991; doi:10.1186/scrt206)

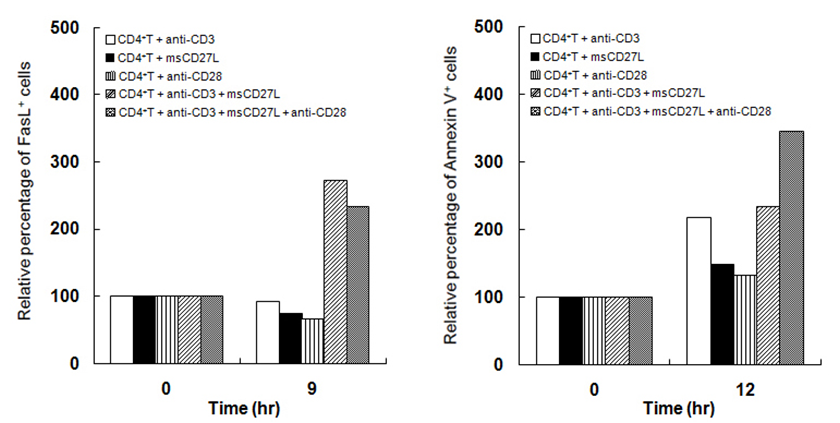

Supplement: Additional file 3 — a figure showing FasL expression on CD4+ T cells (left) and apoptosis of CD4+ T cells (right) increased by anti-CD3 (OKT-3, 1 μg/ml; eBioscience, San Diego, CA, USA) and anti-CD28 (CD28.2, 1 μg/ml; BD Pharmingen, San Diego, CA, USA) agonistic antibodies, and recombinant CD27L (1 μg/ml; R&D Systems) treatment (no shading, CD4+ T + anti-CD3; solid shading, CD4+ T + msCD27L; vertical shading, CD4+ T + anti-CD28; diagonal shading, CD4+ T + anti-CD3 + msCD27L; diagonal crosshatch, CD4+ T + anti-CD3 + msCD27L + anti-CD28). The experiment shown is representative of three independent experiments with three different blood donors. [file scrt206-S3.tiff]

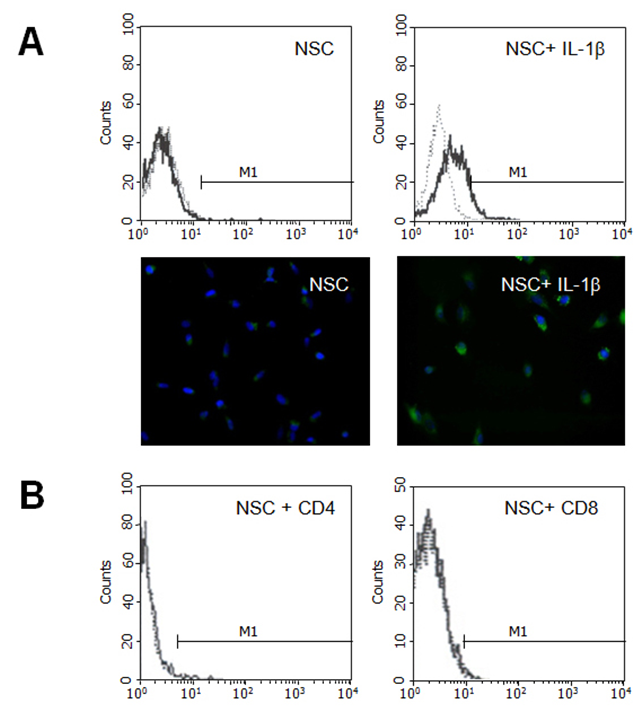

Supplement: Additional file 4 — a figure showing expression of FasL on co-cultured NSCs with allogeneic T cells was examined using FACS analysis (NOK-1) and immunocytochemistry (G247-4). (A) NSCs constitutively did not express FasL. We were able to check the expressions of FasL after IL-1β treatment on NSCs (positive control). (B) FasL expression on co-cultured NSCs with allogeneic T cells was not detected. [file scrt206-S4.tiff]

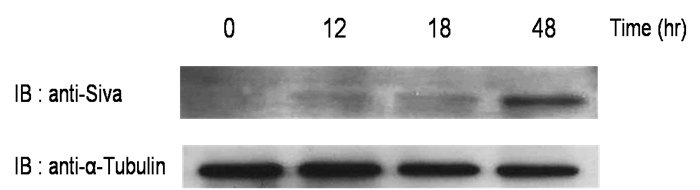

Supplement: Additional file 5 — a figure showing expression of Siva on co-cultured T cells with NSCs. Co-cultured CD4+ T-cell lysate was tested with anti-Siva antibodies (clone C-20; Santa Cruz, CA, USA) by western blotting. α-tubulin was used as a loading control. [file scrt206-S5.tiff]
